# Supplementary material for: Anticancer properties of peptides and protein hydrolysates derived from Asian water monitor (Varanus salvator) serum
Source: PLoS One. 2025 Apr 17;20(4):e0321531. doi: 10.1371/journal.pone.0321531 (PMC12005536; doi:10.1371/journal.pone.0321531)
Supplement: S1 Table — (PDF) [file pone.0321531.s001.pdf]

S1 Table. Inhibitory effect on 18 types of culture cell lines and Vero cells (control) of native peptides derived from serum of *Varanus salvator* (VS) (n=21)

VS No.	Cell lines																		
	A 375	Ca CO2	CAL 27	NCI -H 460	Ha CaT	HeLa	HCT 8	HT29	HepG2	KATO III	MCF -7	MDA -MB- 231	MRC 5	Raw 264.7	SKO V-3	SW 620	T47 D	U937	Vero
	% inhibition																		
1	48.7	83.2	100.8	0.0	88.7	36.1	0.0	69.5	82.0	50.8	97.3	20.2	89.3	60.6	37.2	26.3	22.3	101.9	1.3
2	77.6	106.4	101.8	0.0	98.4	38.1	2.6	86.0	97.2	68.4	117.5	50.9	90.5	61.0	74.1	72.9	50.9	103.0	13.3
3	33.1	87.8	99.6	0.0	93.9	2.5	0.0	79.1	80.3	51.8	92.8	11.9	89.9	75.8	45.6	18.3	30.6	103.2	4.6
4	51.0	78.0	99.4	0.0	93.4	3.0	0.0	75.5	72.3	47.1	93.1	18.9	87.4	75.0	35.6	21.7	35.0	103.5	3.2
5	56.8	94.8	100.3	0.0	92.4	51.2	0.0	75.9	85.9	56.5	101.1	31.1	94.8	53.4	43.4	50.6	37.8	101.6	2.3
6	74.3	89.9	99.6	0.0	95.7	26.4	0.0	81.4	86.6	58.0	101.4	37.2	90.6	59.7	52.9	52.6	40.9	96.8	7.8
7	52.5	82.1	97.3	0.0	94.9	6.3	0.0	76.5	77.8	52.9	79.8	12.7	87.0	61.4	38.7	37.2	28.6	97.5	5.1
8	56.6	84.5	98.1	0.0	89.9	1.1	0.0	72.2	62.2	39.1	75.3	12.7	88.3	77.7	24.8	25.8	29.3	97.6	4.9
9	29.8	101.4	101.5	0.0	97.6	16.8	0.0	83.4	90.7	57.3	101.5	23.8	95.0	64.9	56.8	30.0	48.9	99.1	7.7
10	54.8	91.8	99.3	0.0	84.4	0.0	0.0	87.6	84.3	56.5	78.6	6.5	90.1	92.0	38.1	2.8	31.9	96.5	6.9
11	72.7	107.1	101.9	0.0	99.3	55.3	0.0	87.8	95.3	80.6	119.9	37.7	93.7	72.5	76.5	59.0	60.6	92.7	16.5
12	73.8	104.7	101.5	0.0	99.2	58.1	79.0	99.1	103.5	76.6	119.0	82.3	102.2	96.5	70.4	71.4	84.6	98.4	11.9
13	54.4	107.1	100.4	0.0	97.4	42.3	0.0	85.2	92.6	68.6	113.0	30.6	89.9	64.4	73.0	64.5	45.4	100.5	13.9
14	58.7	102.5	100.9	0.0	96.5	33.4	0.0	81.9	87.8	63.5	111.6	37.8	94.1	61.7	73.1	68.6	47.3	101.6	10.0
15	42.4	103.2	100.1	0.0	94.0	7.4	0.0	76.3	82.7	49.1	94.4	14.4	90.5	60.6	48.8	34.8	44.4	104.1	4.6
16	58.3	103.2	100.4	0.0	97.1	29.7	0.0	74.1	73.3	64.7	104.5	37.3	92.4	66.6	63.8	56.8	51.3	103.6	8.6
17	53.0	102.5	100.7	0.0	95.3	25.2	0.0	77.5	83.6	60.7	104.4	32.4	89.1	59.0	71.8	64.9	46.0	99.8	17.9
18	69.0	106.2	101.1	0.0	94.2	30.7	0.0	87.3	89.5	71.7	104.1	40.4	90.0	69.3	64.7	70.9	51.1	96.6	16.0
19	66.3	106.4	100.6	0.0	97.4	28.5	0.0	88.0	81.9	68.6	104.8	34.8	91.7	65.2	69.3	57.3	54.3	95.1	11.4
20	56.9	99.3	99.5	0.0	96.2	24.6	1.9	87.1	77.6	56.4	99.8	27.8	90.8	62.6	50.6	38.1	42.6	93.6	8.6
21	60.2	99.5	99.9	0.0	90.0	0.0	0.0	84.5	79.1	51.0	73.8	10.9	91.5	72.3	42.3	19.4	38.1	99.3	5.5
Median	56.8	101.4	100.4	0.0	95.3	26.4	0.0	81.9	83.6	57.3	101.4	30.6	90.5	64.9	52.9	50.6	44.4	99.3	7.8
SD	12.6	9.5	1.2	0.0	3.8	18.7	17.2	7.0	9.2	10.3	13.7	17.1	3.3	10.7	15.8	21.1	13.5	3.4	4.8
