# Supplementary material for: Anticancer properties of peptides and protein hydrolysates derived from Asian water monitor (Varanus salvator) serum
Source: PLoS One. 2025 Apr 17;20(4):e0321531. doi: 10.1371/journal.pone.0321531 (PMC12005536; doi:10.1371/journal.pone.0321531)
Supplement: S4 Table — (PDF) [file pone.0321531.s004.pdf]

**S4 Table.** Inhibitory effect on 18 types of culture cell lines and Vero cells of C18 Unbound fraction (hydrophilic) derived from serum of *Varanus salvator* (VS) (n=16)

| VS No. | Cell lines | | | | | | | | | | | | | | | | | | |
|---|---|---|---|---|---|---|---|---|---|---|---|---|---|---|---|---|---|---|---|
| | A 375 | Ca CO2 | CAL 27 | NCI -H 460 | Ha CaT | HeLa | HCT8 | HT29 | HepG 2 | KAT O III | MCF -7 | MDA -MB-231 | MRC 5 | Raw 264.7 | SKO V-3 | SW 620 | T47D | U937 | Vero |
| | % inhibition | | | | | | | | | | | | | | | | | | |
| 1 | 79.7 | 95.4 | 93.9 | 91.4 | 93.6 | 89.4 | 30.8 | 89.1 | 96.6 | 34.6 | 97.4 | 78.9 | 94.9 | 96.5 | 41.3 | 61.1 | 94.3 | 93.8 | 96.4 |
| 2 | 79.6 | 96.5 | 93.5 | 75.2 | 94 | 89.9 | 13.9 | 46.6 | 97.1 | 38 | 97.3 | 85 | 94.9 | 96.3 | 41.7 | 67.2 | 88.4 | 95.3 | 94.7 |
| 3 | 92.5 | 95.2 | 94.8 | 94.6 | 96.7 | 92.1 | 75.5 | 93.4 | 97 | 89 | 95.8 | 92.4 | 96 | 96.8 | 77.8 | 91.8 | 97.2 | 96.2 | 96.9 |
| 4 | 71.7 | 97.7 | 91.1 | 12.9 | 92.3 | 88.8 | 7.4 | 14 | 90.2 | 12.6 | 88.7 | 84 | 92.8 | 92.8 | 46.4 | 24.2 | 80.1 | 96.8 | 91.6 |
| 5 | 87 | 99 | 95.3 | 84 | 94.5 | 92.6 | 33.6 | 70.7 | 98.2 | 90.5 | 96.9 | 86.8 | 97.1 | 97.4 | 51.5 | 68.8 | 93.8 | 96.6 | 95.6 |
| 6 | 95.6 | 97.2 | 96.2 | 96 | 99 | 95.4 | 92.3 | 95.3 | 99 | 96.7 | 97.2 | 91.8 | 97.7 | 97.6 | 81.3 | 95.4 | 98.3 | 96.4 | 98.1 |
| 7 | 64.7 | 99 | 86.2 | 0.1 | 92 | 89.7 | 0 | 7.1 | 41.3 | 2.6 | 43.1 | 85 | 90.3 | 87.4 | 36.6 | 13.1 | 20.4 | 98.4 | 88.1 |
| 8 | 55 | 99.7 | 83.5 | 0 | 89.2 | 90 | 0 | 2.8 | 46.3 | 0 | 22.6 | 82.7 | 96.1 | 78.1 | 24.7 | 0 | 10.7 | 100.4 | 88.1 |
| 9 | 62.8 | 97.1 | 87.3 | 6.6 | 92.4 | 85.4 | 6.1 | 12.5 | 42.5 | 19.1 | 52.1 | 77.8 | 90.8 | 89.5 | 35.3 | 31.3 | 12.7 | 95.9 | 80.2 |
| 10 | 60.1 | 98.6 | 88.1 | 0.9 | 92 | 83.9 | 0 | 0 | 26.7 | 6.2 | 53.5 | 82.4 | 91.5 | 88.3 | 41.7 | 0.9 | 21 | 97.6 | 87.9 |
| 11 | 13.4 | 100.6 | 87.5 | 0 | 71.8 | 54.2 | 0 | 6.8 | 27.4 | 0 | 0 | 79.6 | 88.6 | 33.1 | 37.2 | 0 | 0 | 99.3 | 53.6 |
| 12 | 61 | 100.1 | 83.9 | 2.3 | 81.4 | 82.2 | 0 | 2.4 | 59.4 | 5.1 | 0 | 84.9 | 92.8 | 68.7 | 34.9 | 0 | 0 | 98.7 | 84.7 |
| 13 | 52.6 | 99.2 | 81.3 | 0 | 76.9 | 82 | 0 | 3.7 | 64.4 | 7.3 | 0 | 62.7 | 88.9 | 66.2 | 31.5 | 0.2 | 0 | 99.1 | 67.9 |
| 14 | 80.1 | 99.7 | 93 | 17.1 | 93.1 | 91.8 | 1.6 | 10.8 | 96.8 | 28.4 | 95.9 | 81.1 | 95.5 | 96 | 47.3 | 49.3 | 62.1 | 99.3 | 92.1 |
| 15 | 29.8 | 100.3 | 85.6 | 0 | 34.9 | 56.9 | 0 | 1.5 | 42.5 | 3.5 | 0 | 82.5 | 90.1 | 69.2 | 34.4 | 0 | 0 | 100.3 | 76.5 |
| 16 | 62.9 | 100.3 | 85.5 | 0 | 86 | 83.3 | 6.1 | 5.3 | 58.9 | 3.2 | 4.3 | 78.8 | 94.6 | 77.5 | 35.4 | 8.1 | 0 | 100.4 | 82.6 |
| Median | 63.80 | 99.00 | 87.80 | 4.45 | 92.15 | 89.10 | 3.85 | 8.95 | 61.90 | 9.95 | 52.80 | 82.60 | 93.70 | 88.90 | 39.25 | 18.65 | 20.70 | 98.00 | 88.10 |
| SD | 21.72 | 1.75 | 4.78 | 41.05 | 15.51 | 11.88 | 28.47 | 36.69 | 27.94 | 34.31 | 42.73 | 6.70 | 2.96 | 17.41 | 15.44 | 35.02 | 42.59 | 2.00 | 11.94 |
