# Supplementary material for: Anticancer properties of peptides and protein hydrolysates derived from Asian water monitor (Varanus salvator) serum
Source: PLoS One. 2025 Apr 17;20(4):e0321531. doi: 10.1371/journal.pone.0321531 (PMC12005536; doi:10.1371/journal.pone.0321531)
Supplement: Attachment — Submitted filename: response to reviewers.docx [file pone.0321531.s006.docx]

Dear reviewers

According to some points raised by the academic reviewers to the manuscript entitled “Anticancer Properties of Peptides and Protein hydrolysates Derived from Asian Water Monitor (*Varanus salvator*) serum”. Please see the responses (the answers in blue) to each point below.

Thank you to the reviewers for highlighting the questions. We sincerely appreciate the reviewer’s feedback, as it has assisted us in improving the clarity and completeness of our data presentation and conclusions.

Your sincerely,

Jitkamol Thanasak, Corresponding author

**Reviewer #1:**

1. The manuscript does not provide sufficient evidence to explain the molecular mechanisms by which the peptides exert their anticancer effects. Mechanistic studies, such as those exploring apoptosis induction, cell cycle arrest, or the modulation of key signaling pathways (e.g., PI3K/AKT, MAPK), are necessary to move beyond descriptive findings and contribute to a deeper scientific understanding.

2. The study identifies 159 peptides with potential anticancer activity based on computational predictions but lacks thorough experimental validation. High-confidence predictions must be supported by biochemical assays, such as binding affinity measurements or functional tests in cancer models, to ensure credibility and reliability.

**Answer: 1 & 2**

In this study, we do *in vitro* experiments to screen the cancer inhibitory potential between crude peptides and protein hydrolysates. In addition, the C18 unbound fraction and C18 bound fraction were also used to test (*in vitro*) the anticancer cell growth. Since unbound fraction toxic to Vero cells, we search candidate from C18 bound by performing virtual (*in silico*) screening at the end of this study. **(See the flowchart showing the scope of study)**

The peptides synthesized, modified and subsequently studied *in vitro* to identify the most effective ACPs with less toxicity to Vero cells, as well as molecular biology and/or biochemical assays will be our next chapter. **(Line 305-308, 315-316)**

**Remark: All corrections are printed in blue in the manuscript.**

**The scope of study**

**Reviewer #2:**

This manuscript explores the anticancer potential of crude peptides and protein hydrolysates from Varanus salvator serum across various cancer cell lines. It demonstrates promising inhibitory activity, especially from hydrophilic peptides, with computational insights suggesting novel anticancer peptides. While intriguing, further studies on specificity, safety for normal cells, and peptide optimization are crucial for therapeutic application. On reviewing the manuscript, I found the study to be interesting and potentially suitable for acceptance. However, the manuscript cannot be accepted in its current form. Major revisions are required before it can be considered for acceptance.

1. The manuscript requires improvement in terms of language and grammar. There are several instances of unclear phrasing, and grammatical errors that hinder readability

**Answer:** After all content has been updated based on feedback. The manuscript was checked for consistency, including some grammar improvements.

2. The introduction should include a paragraph discussing the current state of cancer and its related mortality rates.

**Answer:** The content has been updated according to the comments. **(Line 42-49)**

3. The authors must discuss the future prospects and limitations of their study.

**Answer:** The content has been updated according to the comments. **(Line 305-308, 315-316)**

4. In the introduction section, the authors are advised to include the following references to support their statements and provide a stronger foundation for their study.

https://doi.org/10.1016/j.ejmech.2024.117088

https://doi.org/10.1002/slct.202101853

https://doi.org/10.1039/C3RA45749D

https://doi.org/10.1016/j.phymed.2024.155972

https://doi.org/10.3389/fphar.2023.1168566

https://doi.org/10.3389/fphar.2023.1231450

**Answer:** The content has been updated according to the comments. **(Line 64-68)**

**Remark: All corrections are printed in blue in the manuscript.**

**Reviewer #3:**

Manuscript Number: PONE-D-24-53048 Anticancer Properties of Peptides and Protein hydrolysates Derived from Asian Water Monitor (Varanus salvator) serum

The concept of “SAFE” needs explanation. Are Vero cells the test control for “SAFE/ SAFETY”?

37 ….they are safe for normal cells….

**Answer:** The sentence was changed to “ … with less toxicity to Vero cells” **(Line 37)**

82 ….peptides are safe drugs….

**Answer:** This part of the message was discarded. **(Line 97)**

304 ….maintain its safety in normal….

**Answer:** The sentence was changed to “Further modifications may be required to enhance the anticancer efficacy and reduce toxicity to Vero cells.” **(Line 315-316)**

The concept of “normal cell” must be clarified. Are “normal” and/or “control” a non-cancerogenic cell? Vero cells are a continuous cell line, a kind of immortalized cells, but are not Vero “cancer cells”?

HeLa is an immortalized cancer cell line.

The cell line MRC is in the text as cancer cell but in the Lines 127, 128, 209, and 241 is ….MRC-5 = Homo sapiens, NORMAL, Lung.…

205/237 ….HaCaT = Homo sapiens, keratinocyte….

Is keratinocyte cancer or normal cell?

**Answer:** Thank you for your note. HeLa is cervical adenocarcinoma cell line. HaCaT (keratinocyte) and MRC-5 (lung cells) are normal cells. I have already regrouped and published all cell lines in Materials and Methods section **(Line 150-158)**. The rest of the manuscripts were all revised according to this situation.

268 ….the peptides were purified and studied IN SILICO

Definition of IN SILICO.

Serum samples from Varanus salvator were ultra filtered with 3 kDa membrane with and without purification by reverse phase chromatography in C18, peptides tested with cancer and normal cells. I think this is “in vitro”.

**Answer:** An *in silico* experiment had been performed on a computer where the peptide sequences were submitted to web server for anticancer virtual screening. The predicted ACPs were %inhibition. Then the selected peptide sequences were submitted to computer software for peptide folding simulation.

Further study will focus on the top twentieth ACPs. Those will be synthesized and studied *in vitro*.

Table 1, lines 203 to 213 and Table 2, l7774ines 235 to 245 are many repetitions, that explanation could be in the Material and Methods, as a table.

Homo sapiens has 32 repetitions!

Vero cells (control) have 9 repetitions.

colon, colorectal adenocarcinoma; 8 repetitions

adenocarcinoma 22 repetitions

**Answer:** The cell lines were grouped and declarations were moved to the Materials and Methods section. **(Line 150-158)**

Table 1 and Figure 1 have some difference in the numbers. The Figure 1 do not need the numbers, they are represented graphically, and the important values are in the Table 1. Figure 1 has many numbers, it is polluted.

**Answer:** Value numbers in the Figure 1 were discarded. **(Fig 1)**

Lines 214 to 217, is Raw264.7 inhibited by the peptides? The limit is 70% inhibition, Raw264.7 has 64.9. In Figure 1, Raw264.7 is under dashed line 70%.

**Answer:** The 70% cutoff used to determine whether cell inhibition is considered significant. Based on the data presented in the numerical data table showing Raw264.7 (Table 1), we observed an inhibition of Raw264.7 cells at approximately 64.9%, which is below the 70% threshold. Consequently, these cells do not meet the specified inhibition criterion. We understand there may be some confusion when examining the box-plot figure (Fig 1), which includes a dashed line indicating 70%. Nonetheless, the Raw264.7 data point also falls below this line, showing that there is no discrepancy between these two data presentations.

Therefore, we would like to clarify the following points: (1) for Raw264.7 cells, the 64.9% inhibition rate does not surpass the 70% threshold, (2) the data in both the Table 1 and the Figure 1 are consistent, and (3) we will revise the manuscript to explicitly state that Raw264.7 cells do not reach the 70% level, ensuring readers can accurately interpret these findings. **(Fig 1) (Line 230-232)**

91 Materials and Methods

92 Animals and sampling

103 .... centrifuged at 2,500 RPM for 10 ...

RPM units convert to centrifugation speed (× g)

**Answer:** 2,500 RPM was converted to centrifugation speed 1,526 x g. **(Line 118)**

106 Preparation of crude peptides and protein hydrolysate

107 .... fivefold with 0.5 M sodium acetate and….

Sodium acetate pH??

108 ….(Vivaspin 20, GE Healthcare, Chicago, USA)….

(Vivaspin 20 spin columns, Cytiva)

It is necessary indication of centrifugation speed (× g), time, temperature °C,

range of applied diluted sample volume.

112 and 113 Protein hydrolysis: buffer conditions, pH, composition,

concentration, and specific activity of pepsin for hydrolysis.

Sigma–Aldrich has pepsin from ≥400 up to ≥3,200 U/mg protein.

**Answer:** The content has been updated according to the comments. **(Line 122-133)**

116 Peptide Purification by Reverse-Phase Chromatography

The peptides samples were applied without any adjustment, preparation? Like centrifugation or micro filtration? addition of concentrate trifluoroacetic acid?

Volume or protein amount of native and hydrolysate peptide fraction applied to the hydrophobic Delta-Pak C18.

122 ….with 0.1% TFA in acetonitrile…. % acetonitrile? linear or step gradient?

123 & 124 ….Unbound and bound fractions… were evaluated for their anticancer activity.

The fraction from C18 were used directly with Vero and cancer cells without any treatment? pH adjustment? Elimination of acetonitrile? Protein concentration or dilution? Buffer change? Storage condition of the fraction?

**Answer:** The content has been updated according to the comments. **(Line 135-142)**

125 Anticancer activities of the crude peptides and protein hydrolysate

There are not explanations for the condition of the purified fraction in the cytotoxicity test: protein concentration, buffer etc.

**Answer:** The content has been updated according to the comments. **(Line 144-158)**

139 LC‒MS analysis

140 The hydrophobic fractions …. were hydrophilic fractions (bound to C18) analysed by LC-MS analysis?

145 ~154 It is too long, reduce.

**Answer:** The content has been updated according to the comments. **(Line 169-181)**

183 Results

184/185 ….protein hydrolysates….produced some results in cancer cell lines.

Table I showed that protein hydrolysates HAVE NOT INHIBITORY EFFECT over cancer cells. Nine cancer cell lines have 0% inhibition, and the best inhibition effect has only 23,6%.

**Answer:** The content has been updated according to the comments. **(Line 209-220)**

225 By reverse-phase chromatography, the PEPTIDES were purified.

230 Table 2 there are not indication the origin of the pure hydrophobic and hydrophilic after the RP-HPLC Delta-Pak C18.

Lines 117/118 Both the <3 kDa native peptide fraction and the <3 kDa hydrolysate fraction were purified by reverse-phase high-performance liquid chromatography (RP-HPLC)

Table 2 needs separation of the results from native peptide and hydrolysate fraction post-C18. Protein hydrolysate fraction has not anticancer activity but its hydrophilic (C18 unbound) may be has.

**Answer:** Because native peptides have greater anticancer efficiency, so the <3 kDa native peptide fraction were purified and tested (in *vitro*). Peptide Purification by Reverse-Phase Chromatography in Materials and Methods section was edited.

Hydrophilic peptides have inhibitory activity against cancer cells, but also hydrophilic peptides have an effect, 88.1% inhibition, against Vero normal cells!

The test for safety with Vero cells showed that hydrophilic peptides are inhibitors for this line cell.

Anticancer drugs must be toxic for tumoral cells but atoxic for normal cells.

It is confusing the purified peptide fraction origin of Table 2, crude peptides or hydrolyzed protein.

**Answer:** Only <3 kDa native peptide fraction were purified. Hydrophilic peptides have better anticancer efficacy with high toxicity to normal cells. Hydrophobic peptides, on the other hand, appear safer as they have lower anticancer activity. So, LC-MS and in silico were performed, the peptides prediction results will save our work for the next episode.

247 The prediction of ACHP activity showed that 159 of the 432….

It is written in line 140 The HYDROPHOBIC FRACTIONS of both the <3 kDa native peptide and the <3 kDa hydrolysate….LC-MS analysis

The hydrophobic fractions of C18 are not active against cancer cells, Lines 31/32; 227/228 and 268/269/270.

**Answer:** The contrary prediction results (studied *in silico*) vs experimental results (studied *in vitro*) are discussed, which guiding us for the next in vitro study plan. The discussion was edited.

268 ….Hence, the crude/natural peptides were purified and studied in silico “in vitro”.

In the manuscript is not clear the flow of serum proteins, peptides, flow of raw or purified samples to in vitro test like cell growth inhibition, sample fraction purify by C18, purified fraction that are analysed by LC‒MS analysis.

**Answer:** The content has been updated according to the comments. **(Line 276-282) (See the flowchart showing the scope of study)**

272 Based on the calculations, several ACPs exhibited high hydrophobicity

Your experimental data showed that hydrophilic peptides are better for inhibitory activities with cancer cells

**Answer:** The predicted ACPs exhibited higher hydrophobicity, whereas the crude peptides were hydrophilic. Therefore, we plan to synthesize selected peptides and conduct *in vitro* studies.

**Remark: All corrections are printed in blue in the manuscript.**

**The scope of study**
